# Dysregulation of the Intestinal Microbiome in Patients With Haploinsufficiency of A20

**DOI:** 10.3389/fcimb.2021.787667

**Published:** 2022-01-28

**Authors:** Etsushi Toyofuku, Kozue Takeshita, Hidenori Ohnishi, Yuko Kiridoshi, Hiroaki Masuoka, Tomonori Kadowaki, Ryuta Nishikomori, Kenichi Nishimura, Chie Kobayashi, Takasuke Ebato, Tomonari Shigemura, Yuzaburo Inoue, Wataru Suda, Masahira Hattori, Tomohiro Morio, Kenya Honda, Hirokazu Kanegane

**Affiliations:** ^1^ Department of Pediatrics and Developmental Biology, Graduate School of Medical and Dental Sciences, Tokyo Medical and Dental University (TMDU), Tokyo, Japan; ^2^ Graduate School of Medicine and Faculty of Medicine, The University of Tokyo, Tokyo, Japan; ^3^ Department of Microbiology and Immunology, Keio University School of Medicine, Tokyo, Japan; ^4^ Department of Pediatrics, Gifu University Graduate School of Medicine, Gifu, Japan; ^5^ JSR-Keio University Medical and Chemical Innovation Center (JKiC), JSR Corporation, Tokyo, Japan; ^6^ Laboratory for Microbiome Sciences, RIKEN Center for Integrative Medical Sciences, Yokohama, Japan; ^7^ Department of Pediatrics, Kyoto University Hospital, Kyoto, Japan; ^8^ Department of Pediatrics and Child Health, Kurume University School of Medicine, Kurume, Japan; ^9^ Department of Pediatrics, Yokohama City University Graduate School of Medicine, Yokohama, Japan; ^10^ Department of Child Health, Faculty of Medicine, University of Tsukuba, Tsukuba, Japan; ^11^ Department of Pediatrics, Kitasato University Hospital, Sagamihara, Japan; ^12^ Department of Pediatrics, Shinshu University School of Medicine, Matsumoto, Japan; ^13^ Department of Allergy and Rheumatology, Chiba Children’s Hospital, Chiba, Japan; ^14^ Graduate School of Advanced Science and Engineering, Waseda University, Tokyo, Japan; ^15^ Department of Child Health and Development, Graduate School of Medical and Dental Sciences, Tokyo Medical and Dental University (TMDU), Tokyo, Japan

**Keywords:** haploinsufficiency of A20, intestinal microbiome, regulatory T cells, inborn errors of immunity, *Streptococcus mutans*, *Lactobacillus salivarius*

## Abstract

**Introduction:**

Haploinsufficiency of A20 (HA20) is a form of inborn errors of immunity (IEI). IEIs are genetically occurring diseases, some of which cause intestinal dysbiosis. Due to the dysregulation of regulatory T cells (Tregs) observed in patients with HA20, gut dysbiosis was associated with Tregs in intestinal lamina propria.

**Methods:**

Stool samples were obtained from 16 patients with HA20 and 15 of their family members. Infant samples and/or samples with recent antibiotics use were excluded; hence, 26 samples from 13 patients and 13 family members were analyzed. The 16S sequencing process was conducted to assess the microbial composition of samples. Combined with clinical information, the relationship between the microbiome and the disease activity was statistically analyzed.

**Results:**

The composition of gut microbiota in patients with HA20 was disturbed compared with that in healthy family members. Age, disease severity, and use of immunosuppressants corresponded to dysbiosis. However, other explanatory factors, such as abdominal symptoms and probiotic treatment, were not associated. The overall composition at the phylum level was stable, but some genera were significantly increased or decreased. Furthermore, among the seven operational taxonomic units (OTUs) that increased, two OTUs, *Streptococcus mutans* and *Lactobacillus salivarius*, considerably increased in patients with autoantibodies than those without autoantibodies.

**Discussion:**

Detailed interaction on intestinal epithelium remains unknown; the relationship between the disease and stool composition change helps us understand the mechanism of an immunological reaction to microorganisms.

## Introduction

A20, which is encoded by the tumor necrosis factor alpha-induced protein 3 (*TNFAIP3*) gene, is a negative regulator of the TNF-nuclear factor-κB (NF-κB) signaling pathway. Haploinsufficiency of A20 (HA20) causes autoinflammatory and autoimmune disorders (Kadowaki et al., 2018). Dysbiosis of the intestinal microbiome has been observed in some inborn errors of immunities (IEIs)/primary immunodeficiency disease, such as chronic granulomatous disease (CGD) (Sokol et al., 2016), X-linked inhibitor of apoptosis (XIAP) deficiency (Sokol et al., 2016; Ono et al., 2021), tetratricopeptide repeat domain 7A (TTC7A) deficiency (Sokol et al., 2016), common variable immunodeficiency (CVID) (Jørgensen et al., 2016; Varricchi et al., 2021), and Wiskott–Aldrich syndrome (Zhang et al., 2020). Despite IEIs having a monogenetic nature, they vary in severity relating to the microbiome (Liu et al., 2021). Little is known about the mechanism of dysbiosis in patients with IEI, but the association of gut microbiota and regulatory T cells (Tregs) was reported (Liu et al., 2021). Tregs regulate A20 (Kadowaki et al., 2020); as a result, the gut microbiota of patients with HA20 plays a vital role in association with Tregs. Alternatively, the mouse lacking A20 expression in dendritic cells showed intestinal dysbiosis (Talpin et al., 2019). This is the first report of the microbiome in patients with HA20.

## Materials and Methods

### Research Participants and Samples

Kadowaki et al. (2018) previously reported that there were 22 patients with HA20 from nine families in Japan. Thirty-six individuals from nine families, including 18 patients, 17 healthy family members living together, and a sibling whose genetic information is unknown, agreed to participate in this study. Next, fecal samples were obtained from 16 patients and 15 healthy relatives from eight corresponding families. Clinical characteristics of the patients were previously reported (patients 3–5, 7–13, and 17–22), and additional information, such as sex, age at the sample collection, symptoms, and medication, was collected (**Supplementary Table 1**).

### The Classification of the Disease Severity of Haploinsufficiency of A20

We proposed the classification of disease severity for clinical manifestations of patients with HA20. The mild type was defined as asymptomatic or minor phenotype (e.g., mild recurrent stomatitis and/or rash). The moderate type was defined as paroxysmal symptoms (e.g., recurrent fever and/or abdominal pain). The frequency of symptoms is once or more for at least 3 months. The severe type was defined as persistent inflammation (e.g., fever, central nerve lesion, vascular lesion, ocular lesion, intestinal lesion, and/or arthritis) sustained over 2 weeks.

### Detection of the Microbiome

Fecal samples were collected and stored at 4°C under anaerobic conditions until preparation. Fecal samples were stirred until completely homogenized and suspended in phosphate-buffered saline (PBS) containing 20% glycerol (1 g of feces per 5 ml) and poured through a filter to remove large-sized food-derived debris. The fecal suspension was added to EDTA (final concentration of 10 mM, Nacalai) and stored at −80°C until use. After thawing, 100 μl of fecal suspension was gently mixed and incubated in 800 μl of TE10 (10 mM of Tris-HCl and 10 mM of EDTA) buffer containing RNase A (final concentration of 100 μg/ml, Invitrogen) and lysozyme (final concentration of 15 mg/ml, Sigma) for 1 h at 37°C. Purified achromopeptidase (final concentration of 2,000 U/ml, Wako) was added and further incubated for 30 min at 37°C. Sodium dodecyl sulfate (SDS) (final concentration of 1%) and proteinase K (final concentration of 1 mg/ml, Roche) were further added to the mixture and incubated for 1 h at 55°C. High-molecular-weight DNA was extracted with phenol:chloroform:isoamyl alcohol (25:24:1 at pH 7.9), precipitated with isopropanol (equal volume to the aqueous phase), washed with 1 ml of 70% ethanol, and gently resuspended in 30 μl of TE buffer. We used 40 ng of DNA per sample for the sequence.

The sequencing of the 16S ribosomal RNA gene from fecal samples was performed as previously described (Kim et al., 2013; Kakihana et al., 2016). The hypervariable V1–2 region of the 16S rRNA gene was amplified by PCR using barcoded 27Fmod and 338R primers. DNA extracted from *Escherichia coli* Strain SE 11 (isolated from a healthy adult) was used as the positive control template, whereas a DNA-free sample was used as the negative control. The electrophoresis procedure confirmed the PCR amplicons and negative reaction. Then, an equal amount of purified PCR amplicons was sequenced on a MiSeq platform (Illumina, San Diego, CA). Next, 3,000 high-quality reads were randomly selected per sample and analyzed to minimize the overestimation of species richness during clustering associated with the sequencing error (Kim et al., 2013). Good’s coverage index (Singleton et al., 2001) for the 3,000 reads per sample in the current study was 0.980, indicating a high degree of coverage and a sufficient read number for the fecal microbiome analysis. Furthermore, the reads were sorted into operational taxonomic units (OTUs) using the UCLUST algorithm, at a sequence identity threshold of 97% (Stackebrandt and Goebel, 1994; Konstantinidis and Tiedje, 2005). Taxonomic assignments of each OTU were made by similarity searching against the publicly available 16S (RDP v.10.27 and CORE) and National Center for Biotechnology Information genome database, using GLSEARCH. OTU-based microbial diversity was estimated using the Shannon index with Scikit-bio (v.0.5.5). A comparison of each group (family, HA20) in the unweighted and weighted UniFrac principal coordinate analysis (PCoA) was evaluated by using permutational multivariate ANOVA (PERMANOVA) with Scikit-bio (v.0.5.5) (Anderson, 2017).

### Statistical Analysis

Statistical analysis of OTUs and Shannon index was performed using the Wilcoxon rank-sum test. Values are represented as median (minimum–maximum). The threshold for significance was *p* < 0.05. Also, stratified analysis was performed using the two-tailed t-test for ordinal scale or regression analysis for continuous scale. Statistical analyses were conducted using R package (exactRankTests) or JMP Pro v.16.0.0 (SAS Institute Inc., NC, USA). The linear discriminant analysis (LDA) effect size (LEfSe) method was performed on the Huttenhower lab Galaxy server (https://huttenhower.sph.harvard.edu/galaxy/) by importing the bacterial relative abundance values at the genus level with Kruskal–Wallis test *p* < 0.05 and LDA score (log10) > 2 (Segata et al., 2011).

## Results

Thirty-one fecal samples from 16 patients and 15 healthy family members were sequenced. However, samples from three patients who received antibiotic treatment within 4 weeks of stool collection were excluded from statistical analysis. Also, two samples from children under the age of 3 years were excluded from statistical analysis because their gut microbiome is naturally different from the older’s microbiome (Yatsunenko et al., 2012). Nobody but an excluded younger brother (P22-sib) has food allergies. The median (min–max) age of 13 patients with HA20 and their healthy family members of 13 individuals were 19 (3–43) and 36 (4–49) years (*p* = 0.09, Wilcoxon rank-sum test), respectively. Patients with HA20 consist of four mild, four moderate, and five severe patients. Eleven of 13 patients and eight of 13 presented with stomatitis and abdominal symptoms, respectively. One patient was treated with probiotics and 5 with immunosuppressants (**Supplementary Table 1**).

**Figure 1:**
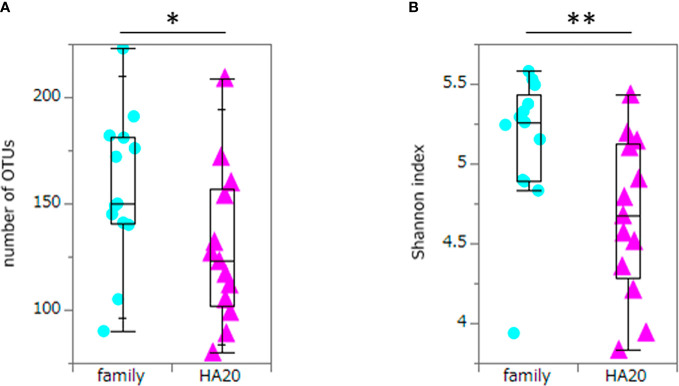
The numbers of OTUs and Shannon index. The numbers of OTUs (median 123 *vs.* 150, *p* = 0.049) **(A)** and Shannon index (median 4.68 *vs.* 5.26, *p* = 0.009) **(B)** of patients with HA20 and their family members. Box plot indicates median value, and lower and upper quartiles. *p*-Value is calculated using the Wilcoxon rank-sum test. *p < 0.05, **p < 0.01. OTU, operational taxonomic unit; HA20, haploinsufficiency of A20.

**Figure 2:**
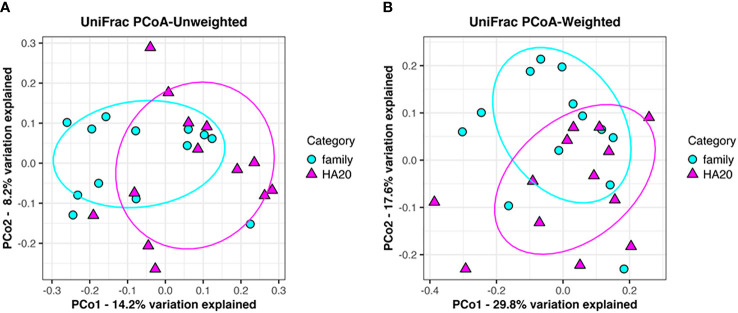
Principal component analysis of gut flora. Clustering of patients (red triangle) and their families (blue circle) following unweighted UniFrac analysis (*p* = 0.008). **(A)** Weighted UniFrac analysis (*p* = 0.059). **(B)** Explained variation is represented as a percentage.

Ten thousand reads were used from an average (and SD) of 53,935 (± 9,383) raw reads. An average of 9,848 reads passed the filter, and every 3,000 Passed Filter reads were used for OTU analysis. The number of OTUs in patients’ samples was considerably less than their healthy members (median 123 *vs.* 150, *p* = 0.049, Wilcoxon rank-sum test) (**Figure 1A**). Likewise, the Shannon index in patients’ samples was significantly less than their healthy members (median 4.68 *vs.* 5.26, *p* = 0.009, Wilcoxon rank-sum test) (**Figure 1B**), whereas the principal component analysis showed a significant difference in the composition of the intestinal microbiome in unweighted UniFrac analysis (*p* = 0.008, PERMANOVA) (**Figure 2A**), although not significant in weighted UniFrac analysis (*p* = 0.059) (**Figure 2B**). Thus, the stratified analysis suggested that any sex, stomatitis, abdominal symptoms, and use of probiotics do not affect the microbiome diversity (**Tables 1** and **2**). Lower age was significantly associated with a lower Shannon index although insignificantly with OTUs. Both disease severity and immunosuppressant use were associated with a lower number of OTUs with significance, and they showed lower Shannon index although not significantly.

**Table 1:** Univariable analysis of Shannon index.

Parameter	Median (IQR) or N (percentage)	Risk difference (95% CIs)	*p*-Value
Age	19 (6.5–33.5)	0.020 (0.0030 to 0.0370)	**0.03**
Sex (male or female)	8 (62%)	0.25 (−0.38 to 0.87)	0.41
Severity (severe or not)	5 (28%)	−0.39 (−0.98 to 0.20)	0.18
Stomatitis	11 (85%)	0.49 (−0.32 to 1.30)	0.21
Abdominal symptoms	8 (62%)	0.18 (−0.46 to 0.81)	0.56
Probiotics	1 (8%)	0.13 (−1.04 to 1.31)	0.81
Immunosuppressants	5 (28%)	−0.18 (−0.82 to 0.45)	0.53

Statistical analyses were performed using the two-tailed t-test for ordinal scale or regression analysis for continuous scale. Bold indicates a significant difference.

IQR, interquartile range.

**Table 2:** Univariable analysis of OTUs.

Parameter	Median (IQR) or N (percentage)	Risk difference (95% CIs)	*p*-Value
Age	19 (6.5–33.5)	0.64 (−0.88 to 2.17)	0.37
Sex (male or female)	8 (62%)	21.5 (−55.3 to 39.2)	0.36
Severity (severe or not)	5 (28%)	−36.3 (−77.3 to 4.7)	**0.04**
Stomatitis	11 (85%)	27.9 (−34.1 to 88.8)	0.35
Abdominal symptoms	8 (62%)	21.2 (−33.5 to 60.0)	0.55
Probiotics	1 (8%)	11.3 (−72.4 to 95.0)	0.61
Immunosuppressants	5 (28%)	−41.0 (−78.0 to −3.9)	**0.02**

Statistical analyses were performed using the two-tailed t-test for ordinal scale or regression analysis for continuous scale. Bold indicates a significant difference.

OTU, operational taxonomic unit; IQR, interquartile range.

**Figure 3:**
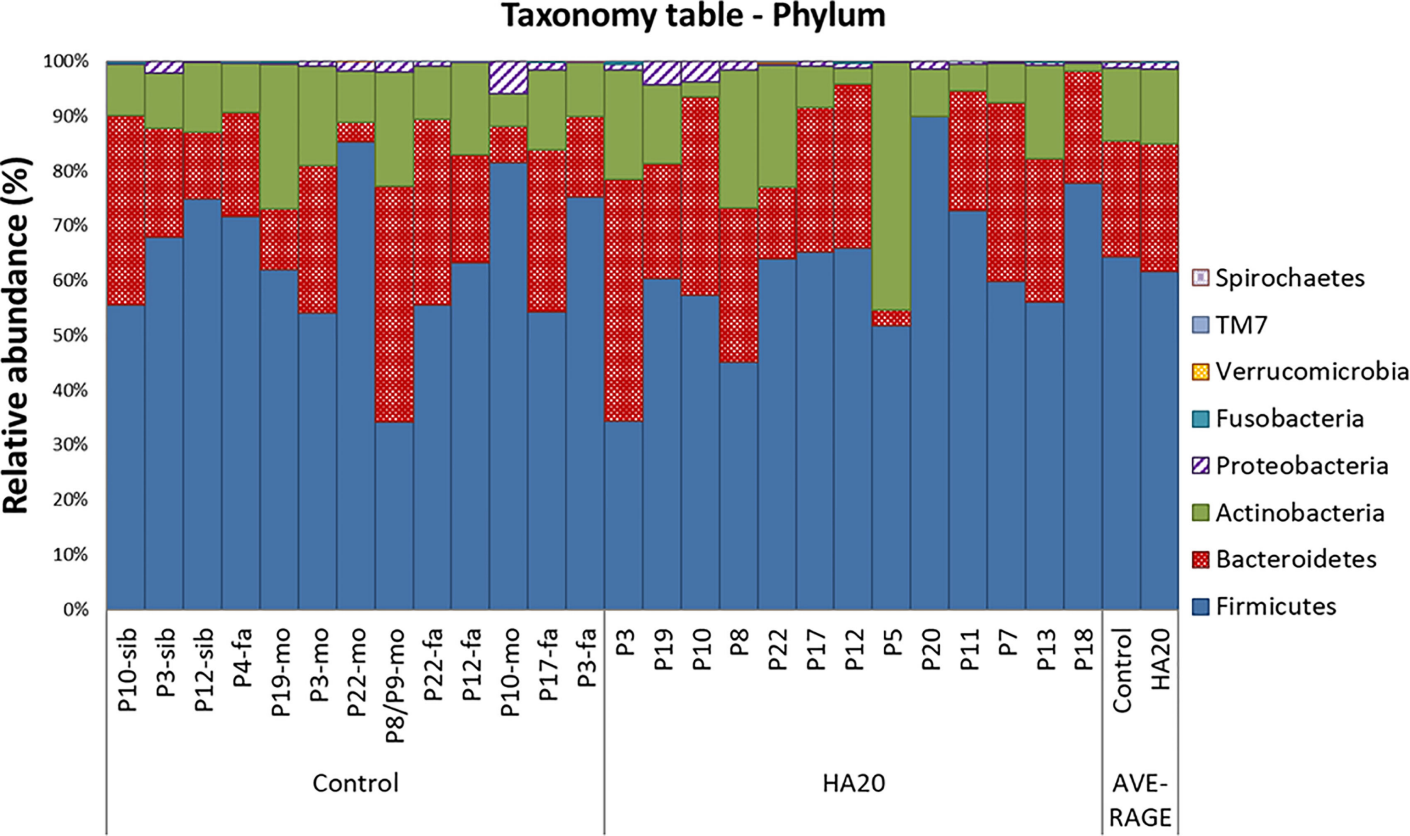
The composition of gut bacterial microbiota. The global composition of bacterial microbiota at the phylum level for the indicated groups. The healthy family members (left) and patients (middle) are represented in order of age. The two bars on the right are average values. P, patient; fa, Father; mo, mother; sib, sibling.

**Figure 4:**
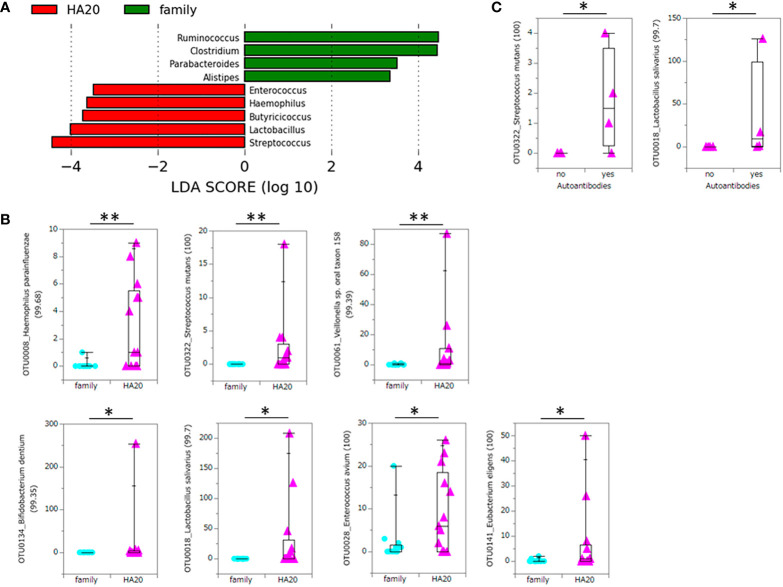
Significant increase or decrease of bacteria at the genus level and increase at the OTU level. The LDA score of patients with HA20 compared with their family members (Kruskal–Wallis test p < 0.05 and LDA score (log10) > 2) are represented. **(A)** The increased OTUs in patients with HA20 compared with their family members. **(B)** and the increased OTUs in patients with autoantibodies compared with those without antibodies. **(C)** Their closest species or strain and percentage similarity (%) in the Ribosomal Database Project (RDP) database are indicated. Box plot indicates the median value, and lower and upper quartiles. p-Values are calculated using the Wilcoxon rank-sum test. *p < 0.05, **p < 0.01. OTU, operational taxonomic unit; LDA, linear discriminant analysis; HA20, haploinsufficiency of A20.

At the phylum level, there was no significant difference between patients with HA20 and their family members (**Figure 3** and **Supplementary Table 2**). Conversely, at the genus level, five genera (*Streptococcus*, *Lactobacillus*, *Butyricicoccus*, *Haemophilus*, and *Enterococcus*) increased, and another four (*Ruminococcus*, *Clostridium*, *Parabacteroides*, and *Alistipes*) decreased in patients with HA20 compared with their family members (LDA score greater than two at *p* < 0.05 with LEfSe analysis) (**Figure 4A**). At the OTU level, seven OTUs significantly increased in patients with HA20 compared with their family members (*p* < 0.001: closest species/strain name *Haemophilus parainfluenzae*, *Streptococcus mutans*, and *Veillonella* sp. oral taxon 158, *p* < 0.05: *Bifidobacterium dentium*, *Lactobacillus salivarius*, *Enterococcus avium*, and *Eubacterium eligens*, Wilcoxon rank-sum test). Most of these OTUs are indigenous bacteria and are undetected in the intestinal microbiota. As a representative, only one of two healthy child siblings under 10 years and none of 11 healthy adult members had *H. parainfluenzae*, when all six child patients with HA20 and two of seven adult patients with HA20 had it (**Figure 4B**). None of these seven OTUs showed a significant correlation with either stomatitis or abdominal symptoms. However, two of seven OTUs showed a significant difference between patients with and without autoantibodies (four and five patients, respectively; four patients were not analyzed) (**Figure 4C**).

## Discussion

This study demonstrated that patients with HA20 show intestinal dysbiosis. Despite HA20 being a genetically occurring disease, previous studies report bacterial composition changes in other IEIs. XIAP deficiency is a rare IEI, and it is characterized by recurrent hemophagocytic lymphohistiocytosis and refractory inflammatory bowel disease (IBD) similar to Crohn’s disease. Furthermore, patients with XIAP deficiency showed intestinal dysbiosis and IBD, which are rescued by allogeneic hematopoietic cell transplantation (Ono et al., 2021). These data further establish the hypothesis that monogenic disease is the cause of gut dysbiosis. From previous studies, there are 118 of 354 IEIs that exhibit gastrointestinal symptoms (Hartono et al., 2019), and the interaction between bacteria and the immune system might vary with the disease. For instance, immunoglobulin A secreted at the mucosal surface of the gastrointestinal tract was associated with selective IgA deficiency (Berbers et al., 2019), and the absence of various subsets of T cells in intestinal lamina propria was associated with X-linked severe combined immunodeficiency (Clarke et al., 2018).

The mechanism that causes dysbiosis in patients with HA20 is mediated by Tregs because it is a key factor of immunoregulatory disorders in patients with HA20 (Liu et al., 2021), and in the mouse model of Treg-associated monogenic autoimmune disorders, *Aire* knockout mouse showed significant dysbiosis (Dobeš et al., 2015). Mouse models, *Aire*-deficient mice (Gray et al., 2007), and *Foxp3* knockout mice (Chinen et al., 2010) exhibit an autoimmune phenotype even in germ-free conditions, and it was concluded that these diseases are independent of commensal microbial regulation (Liu et al., 2021). Conversely, the Treg-depleted model showed more severe inflammation in the small intestine of specific pathogen-free mice than germ-free mice, which indicates that the disease severity is associated with the microbiome (Liu et al., 2021). These findings indicate the possible association between disease severity and gut dysbiosis. Additionally, data from this study showed a significant correlation between disease severity and dysbiosis. The use of immunosuppressants, an alternative indicator of disease severity, also correlated with dysbiosis.

Another possible mechanism of dysbiosis is metabolism. A mouse model lacking A20 in specific dendritic cells reported aberrant expression of antibacterial peptides and luminal dysbiosis (Talpin et al., 2019).

Ten of 18 patients with HA20 were treated with immunosuppressants (Kadowaki et al., 2020), and one refractory patient successfully received hematopoietic cell transplantation (Shiraki et al., 2021). However, less invasive therapy would be required. Successful fecal microbiota transplantation (FMT) is reported in patients with HIV (Serrano-Villar et al., 2021) and the mouse model of Behcet’s disease (Ye et al., 2018), while there is no evidence of FMT in patients with IEIs including HA20. Further study is required to clarify the mechanism of dysbiosis in patients with HA20 and IEIs, to determine the biomarker of the disease, and to discover if FMT works or not.

Several monogenic IEIs inform us how the disease-specific immune dysfunction causes disease-specific dysbiosis. Collectively, we can easily understand how the complex immune systems respond to various microorganisms. This study showed that *S. mutans* and *L. salivarius* significantly increased in both patients with HA20, those with autoantibodies compared with family members and patients without autoantibodies, respectively. Since the sample size was small for statistical analysis (13 *vs.* 13, and 4 *vs.* 5, respectively), statistical significance in two manners was rare enough to reject the null hypothesis. Also, although the close mechanism remains unclear, it was suggested that these two OTUs might be associated with autoimmune onset.

In the context of statistical analysis, we excluded patients younger than 3 years and/or patients with antibiotics use because they present a confounding bias for microbiota (Hopkins et al., 2002; Yatsunenko et al., 2012; Ramirez et al., 2020). However, our data suggested that lower age is the explanatory variable for lower Shannon index, not for OTU numbers. This potential confounding bias is statistically a problem but not clinically crucial. Considering clinical management, both lower age and antibiotics use affect disease severity as well as microbiome diversity. Indeed, the excluded patients based on antibiotics use [previously described as P4, P9, and P21 (Kadowaki et al., 2018)] were relatively younger (4, 1, and 3 years old, respectively) and more severe (P4 and P9 are moderate; P21 is severe) than the included patients. Early-onset patients could be regarded as more strongly influenced by genetics and so potentially more severe than late-onset patients.

This study has several limitations. Firstly, clinical heterogeneity exists among medications, such as probiotics and antibiotics. The potential confounding factor is underestimated because of the small sample size. Secondly, because of rare diseases, it is difficult to collect more fecal samples for statistical analysis of the potential confounding factor described. Multivariate analysis was unfeasible due to small samples. Thirdly, not all patients with HA20 received gastrointestinal endoscopy, and so alternative indicators such as the disease severity or abdominal symptoms do not necessarily reflect the exact intestinal condition.

## Concluding Remarks

We investigated the intestinal microbiome composition in patients with HA20 and identified that seven OTUs significantly increased in the patients. In particular, *S. mutans* and *L. salivarius* significantly increased in patients with HA20 and autoantibodies, and these OTUs were associated with autoimmune onset.

## Data Availability Statement

The datasets for this article are not publicly available as regards participant/patient anonymity. Requests to access the datasets should be forwarded to the corresponding author.

## Ethics Statement

Written informed consent was obtained from patients or their parents. This study was conducted following the Declaration of Helsinki and approved by the ethics boards of Tokyo Medical and Dental University and Keio University.

## Author Contributions

ET, KT, and YK contributed to data management. KT, YK, WS, HM, WS, and MH conducted DNA sequencing and bioinformatics analysis. HO and HK managed sample recruitment. HO, TK, RN, KN, CK, TE, TS, and YI contributed to sample collection. ET, KT, and HK wrote the manuscript. TM and KH provided critical discussion. HK designed and managed the project. All authors read and approved the final manuscript.

## Funding

This work was partly supported by MEXT/JSPS KAKENHI (Grant Number: JP17K10099) and Naoki Tsuchida Research Grant to HK.

## Conflict of Interest

KH is a scientific advisory board member of Vedanta Biosciences and 4BIO CAPITAL. YK is an employee of JSR Corporation.

The remaining author declares that the research was conducted in the absence of any commercial or financial relationships that could be construed as a potential conflict of interest.

## Publisher’s Note

All claims expressed in this article are solely those of the authors and do not necessarily represent those of their affiliated organizations, or those of the publisher, the editors and the reviewers. Any product that may be evaluated in this article, or claim that may be made by its manufacturer, is not guaranteed or endorsed by the publisher.
