# Supplementary material for: Dysregulation of the Intestinal Microbiome in Patients With Haploinsufficiency of A20
Source: Front Cell Infect Microbiol. 2022 Jan 28;11:787667. doi: 10.3389/fcimb.2021.787667 (PMC8834539; doi:10.3389/fcimb.2021.787667)
Supplement: Supplementary file 1 [file DataSheet_1.docx]

**Supplementary Table 1. Characteristics and microbiome diversity of the patients with HA20 and their relatives**

| ID | Sex | Age | Severity | Stomatitis | Abdominal symptoms | Antibiotics use from 6 months to 4 weeks ago | Probiotics within past 4 weeks | Immunosuppressant use | OTU number | Shannon index |
| --- | --- | --- | --- | --- | --- | --- | --- | --- | --- | --- |
| P3-fa | M | 49 | - | - | - | NA | no | no | 182 | 5.24 |
| P3-mo | F | 34 | - | - | - | NA | no | no | 181 | 5.38 |
| P3-sib | F | 6 | - | - | - | NA | no | no | 140 | 4.83 |
| P4-fa | M | 31 | - | - | - | NA | no | no | 172 | 5.33 |
| P8/P9-mo | F | 37 | - | - | - | NA | no | no | 105 | 4.89 |
| P10-mo | F | 42 | - | - | - | NA | no | no | 90 | 3.94 |
| P10-sib | F | 4 | - | - | - | NA | no | no | 149 | 4.90 |
| P12-fa | M | 42 | - | - | - | NA | no | no | 141 | 5.49 |
| P12-sib | M | 14 | - | - | - | NA | no | no | 150 | 5.26 |
| P17-fa | M | 43 | - | - | - | NA | NA | NA | 145 | 5.29 |
| P19-mo | F | 32 | - | - | - | NA | no | no | 191 | 5.15 |
| P19-sib | F | 1 | - | - | - | NA | no | no | * | * |
| P22-fa | M | 37 | - | - | - | NA | no | no | 176 | 5.53 |
| P22-mo | F | 36 | - | - | - | NA | no | no | 223 | 5.58 |
| P22-sib | M | 2 | - | - | - | NA | no | no | * | * |
| P3 | M | 3 | severe | no | yes | CFPN-PI | no | yes; etanercept, MTX, PSL | 80 | 3.83 |
| P4 | F | 4 | moderate | no | yes | PIPC, CAM, TFLX, VRCZ | no | yes: etanercept | † | † |
| P5 | F | 28 | mild | no | yes | no | no | no | 132 | 4.68 |
| P7 | M | 34 | mild | yes | no | no | no | no | 160 | 5.14 |
| P8 | F | 7 | mild | yes | no | no | no | no | 89 | 4.21 |
| P9 | F | 1 | moderate | no | no | PIPC, CTRX, TFLX | yes: Enterococcus faecium, Clostridium butyricum, Bacillus subtilis | no | *† | *† |
| P10 | M | 6 | moderate | yes | no | AMPC | no | yes; MTX | 117 | 4.57 |
| P11 | M | 33 | moderate | yes | yes | NA | no | no | 209 | 5.43 |
| P12 | M | 19 | severe | yes | no | NA | no | yes | 127 | 4.36 |
| P13 | F | 42 | severe | yes | yes | NA | no | yes | 105 | 4.91 |
| P17 | F | 9 | moderate | yes | yes | CDTR-PI | no | no | 172 | 5.19 |
| P18 | F | 43 | severe | yes | yes | ST | no | yes; biological agent | 123 | 5.10 |
| P19 | M | 5 | severe | yes | yes | no | no | no | 99 | 3.94 |
| P20 | M | 33 | mild | yes | no | NA | no | no | 112 | 4.51 |
| P21 | M | 3 | severe | no | yes | FOM | yes: Clostridium butyricum, Bifidobacterium | yes; PSL, CyA | † | † |
| P22 | M | 7 | moderate | yes | yes | no | yes: Clostridium butyricum | no | 154 | 4.79 |

Characteristics and microbiome diversity of the patients and their relatives are shown. The current ID of the patients is the same as the ID at the previous report (1).

Abbreviation: NA, not available; P, patient; fa, Father; mo, mother; sib, sibling; CFPN-PI, Cefcapene Pivoxil; PIPC, Piperacillin; CAM, Clarithromycin; TFLX, Tosufloxacin; VRCZ, Voriconazole; CTRX, Ceftriaxone; AMPC, Amoxicillin; CDTR-PI; Cefditoren Pivoxil; ST, Sulfamethoxazole - Trimethoprim; FOM, Fosfomycin; MTX, methotrexate; PSL, prednisolone; CyA, cyclosporin A.

*excluded from statistical analysis because they were younger than 3 years old

†excluded from statistical analysis because they had received antibiotic agents within 4 weeks of sample collection.

**Supplementary Table 2. The composition of gut microbiota at the phylum level**

| Category | Control | | | | | | | | | | | | | | |
| --- | --- | --- | --- | --- | --- | --- | --- | --- | --- | --- | --- | --- | --- | --- | --- |
| Sample | P10-sib | P3-sib | P12-sib | P4-fa | P19-mo | P3-mo | P22-mo | P8/P9-mo | P22-fa | P12-fa | P10-mo | P17-fa | P3-fa | average | SE |
| Firmicutes | 1664 | 2035 | 2245 | 2149 | 1861 | 1621 | 2557 | 1025 | 1664 | 1897 | 2448 | 1628 | 2256 | 1926.9 | 115.4 |
| Bacteroidetes | 1039 | 599 | 366 | 572 | 333 | 807 | 109 | 1292 | 1020 | 593 | 199 | 888 | 444 | 635.5 | 98.4 |
| Actinobacteria | 280 | 304 | 386 | 267 | 793 | 547 | 280 | 622 | 290 | 505 | 175 | 436 | 295 | 398.5 | 48.5 |
| Proteobacteria | 8 | 62 | 3 | 8 | 7 | 25 | 52 | 60 | 24 | 4 | 178 | 45 | 4 | 36.9 | 13.3 |
| Fusobacteria | 8 | 0 | 0 | 3 | 6 | 0 | 1 | 0 | 1 | 0 | 0 | 3 | 0 | 1.7 | 0.7 |
| Verrucomicrobia | 0 | 0 | 0 | 0 | 0 | 0 | 1 | 0 | 0 | 0 | 0 | 0 | 0 | 0.1 | 0.1 |
| TM7 | 1 | 0 | 0 | 1 | 0 | 0 | 0 | 1 | 1 | 1 | 0 | 0 | 0 | 0.4 | 0.1 |
| Spirochaetes | 0 | 0 | 0 | 0 | 0 | 0 | 0 | 0 | 0 | 0 | 0 | 0 | 1 | 0.1 | 0.1 |

| HA20 | | | | | | | | | | | | | | | *p* value |
| --- | --- | --- | --- | --- | --- | --- | --- | --- | --- | --- | --- | --- | --- | --- | --- |
| P3 | P19 | P10 | P8 | P22 | P17 | P12 | P5 | P20 | P11 | P7 | P13 | P18 | average | SE | Wilcoxon rank sum test |
| 1029 | 1811 | 1720 | 1352 | 1919 | 1955 | 1979 | 1555 | 2698 | 2185 | 1795 | 1681 | 2331 | 1846.9 | 117.5 | 0.71 |
| 1326 | 630 | 1089 | 849 | 395 | 790 | 898 | 82 | 1 | 656 | 982 | 789 | 614 | 700.1 | 103.7 | 0.51 |
| 599 | 430 | 77 | 751 | 667 | 227 | 85 | 1360 | 260 | 146 | 212 | 508 | 45 | 412.8 | 102.7 | 0.44 |
| 32 | 129 | 114 | 48 | 8 | 28 | 26 | 3 | 41 | 12 | 11 | 19 | 10 | 37.0 | 11.1 | 0.62 |
| 14 | 0 | 0 | 0 | 0 | 0 | 12 | 0 | 0 | 0 | 0 | 3 | 0 | 2.2 | 1.3 | 0.43 |
| 0 | 0 | 0 | 0 | 10 | 0 | 0 | 0 | 0 | 0 | 0 | 0 | 0 | 0.8 | 0.8 | 1.00 |
| 0 | 0 | 0 | 0 | 1 | 0 | 0 | 0 | 0 | 1 | 0 | 0 | 0 | 0.2 | 0.1 | 0.38 |
| 0 | 0 | 0 | 0 | 0 | 0 | 0 | 0 | 0 | 0 | 0 | 0 | 0 | 0.0 | 0.0 | 1.00 |

The numbers of OTUs at the phylum level are shown.

The healthy family members (upper) and patients (lower) are represented in order of age.

The representation corresponds to Figure 3.

P, patient; fa, Father; mo, mother; sib, sibling; SE, standard error of the mean.
